# Cost-Effectiveness of BRCA 1/2 Genetic Test and Preventive Strategies: Using Real-World Data From an Upper-Middle Income Country

**DOI:** 10.3389/fonc.2022.951310

**Published:** 2022-07-11

**Authors:** Marina Lourenção, Julia Simões Correa Galendi, Henrique de Campos Reis Galvão, Augusto Perazzolo Antoniazzi, Rebeca Silveira Grasel, André Lopes Carvalho, Edmundo Carvalho Mauad, Jorge Henrique Caldeira de Oliveira, Rui Manuel Reis, Olena Mandrik, Edenir Inêz Palmero

**Affiliations:** ^1^ School of Economics, Business Administration and Accounting at Ribeirão Preto, University of São Paulo, Ribeirão Preto, Brazil; ^2^ Molecular Oncology Research Center, Barretos Cancer Hospital, Barretos, Brazil; ^3^ Institute of Health Economics and Clinical Epidemiology, Faculty of Medicine and University Hospital of Cologne, University of Cologne, Cologne, Germany; ^4^ Early Detection Prevention and Infections, International Agency for Research on Cancer, Lyon, France; ^5^ Life and Health Sciences Research Institute (ICVS), School of Medicine, University of Minho, Braga, Portugal; ^6^ ICVS/3B’s – PT Government Associate Laboratory, Guimarães, Portugal; ^7^ School of Health and Related Research, The University of Sheffield, Sheffield, United Kingdom; ^8^ Department of Genetics, Brazilian National Cancer Institute, Rio de Janeiro, Brazil

**Keywords:** breast cancer, ovarian cancer, *BRCA* genetic test, preventive strategies, cost-effectiveness

## Abstract

Although BRCA1/2 genetic testing in developed countries is part of the reality for high-risk patients for hereditary breast and ovarian cancer (HBOC), the same is not true for upper-middle-income countries. For that reason, this study aimed to evaluate whether the BRCA1/2 genetic test and preventive strategies for women at high risk for HBOC are cost-effective compared to not performing these strategies in an upper-middle-income country. Adopting a payer perspective, a Markov model with a time horizon of 70 years was built to delineate the health states for a cohort of healthy women aged 30 years that fulfilled the *BRCA1/2* testing criteria according to the guidelines. Transition probabilities were calculated based on real-world data of women tested for *BRCA1/2* germline mutations in a cancer reference hospital from 2011 to 2020. We analyzed 275 *BRCA* mutated index cases and 356 *BRCA* mutation carriers that were first- or second-degree relatives of the patients. Costs were based on the Brazilian public health system reimbursement values. Health state utilities were retrieved from literature. The *BRCA1/2* genetic test and preventive strategies result in more quality-adjusted life years (QALYs) and costs with an incremental cost-effectiveness ratio of R$ 11,900.31 (U$ 5,504.31)/QALY. This result can represent a strong argument in favor of implementing genetic testing strategies for high-risk women even in countries with upper-middle income, considering not only the cancer prevention possibilities associated with the genetic testing but also its cost-effectiveness to the health system. These strategies are cost-effective, considering a willingness-to-pay threshold of R$ 25,000 (U$ 11,563.37)/QALY, indicating that the government should consider offering them for women at high risk for HBOC. The results were robust in deterministic and probabilistic sensitivity analyses.

## Introduction

Breast cancer is the most common cancer worldwide and the leading cause of cancer death among women, with globally more than 2.6 million new cases and almost 700,000 deaths annually (1). Despite being less frequent (about 300,000 new cases annually), ovarian cancer has a high lethality rate, with almost seven deaths for every 10 new cases diagnosed (1).

Individuals with hereditary cancer have a higher risk of developing cancer during their lifetime when compared to the general population. Although many high and moderate cancer genes have been discovered and associated with hereditary breast and ovarian cancer (HBOC) in the last years, *BRCA*1 and *BRCA*2 still account for most cases (2–4). Pathogenic germline variants in these genes confer a high risk for developing breast and/or ovarian cancer that can reach 72% and 44% for *BRCA*1 mutation carriers and 69% (for breast cancer) and 17% (for ovarian cancer) for those with *BRCA2* pathogenic alterations (5–7).

Women with personal and/or family history suggestive of HBOC should be referred for genetic counselling and genetic testing to investigate for the presence of pathogenic germline variants (8). Besides, the realization of genetic testing makes it possible to offer it to asymptomatic relatives of the index patient in a predictive context. In this context, the preventive medicine is brought into evidence, once for women who tested positive can be recommended to attend to intensified surveillance for early-detection tumors or risk reduction surgeries, such as risk-reducing bilateral mastectomy (RRBM) and/or risk-reducing salpingo-oophorectomy (RRSO) (2, 9–12). In addition, for relatives not carriers of the pathogenic variant segregating in the family, standard care can be offered (8), once they are not at increased risk of breast or ovarian cancer (13–16).

Several studies on the cost-effectiveness analysis of genetic testing have been performed worldwide. Although previous economic modeling studies indicate that it is cost-effective to provide population-based genetic tests (17–19), the Brazilian Universal Health Coverage System (SUS) does not provide BRCA genetic tests to high-risk women for HBOC. Since 71.5% of the Brazilian population relies exclusively on the SUS (20), most Brazilian women do not have access to the personalized measures for prevention and early diagnosis, as recommended by international guidelines. Thus, this study aimed to evaluate whether offering *BRCA1/2* genetic testing followed by preventive strategies for women at high risk for HBOC is cost-effective when compared to no genetic testing (i.e., and no preventive strategies) in the context of the public health system of an upper middle-income country with continental dimensions as Brazil.

## Methods

We developed a Markov model to assess whether *BRCA1/2* testing and preventive strategies for healthy women at high risk for HBOC are cost-effective compared to standard care (no testing and no preventive strategies). Using the TreeAge Pro, the model estimates the costs and benefits, and the latter is expressed as quality-adjusted life years (QALYs) and life years gained (LYG), highly used in cost-effectiveness studies. To reflect the long-term consequences of breast and ovarian cancer, the model had a 1-year cycle length and time horizon of 70 years (lifetime). The analysis was conducted from the perspective of the SUS as the payer. In line with recommendations from the Brazilian guideline for economic evaluations, costs and effects were discounted at 5% (21).

Considering that HBOC women started annual mammography at 30 years (8) and the low incidence of breast and ovarian cancer at younger ages (22), the target population was a cohort of 30-year-old Brazilian women without a history of breast or ovarian cancer but with first or second-degree relatives who have *BRCA*-related cancer and that fulfilled the National Comprehensive Cancer Network (NCCN) clinical criteria for *BRCA* testing (8).

### Strategies for the Comparison

**Figure 1:**
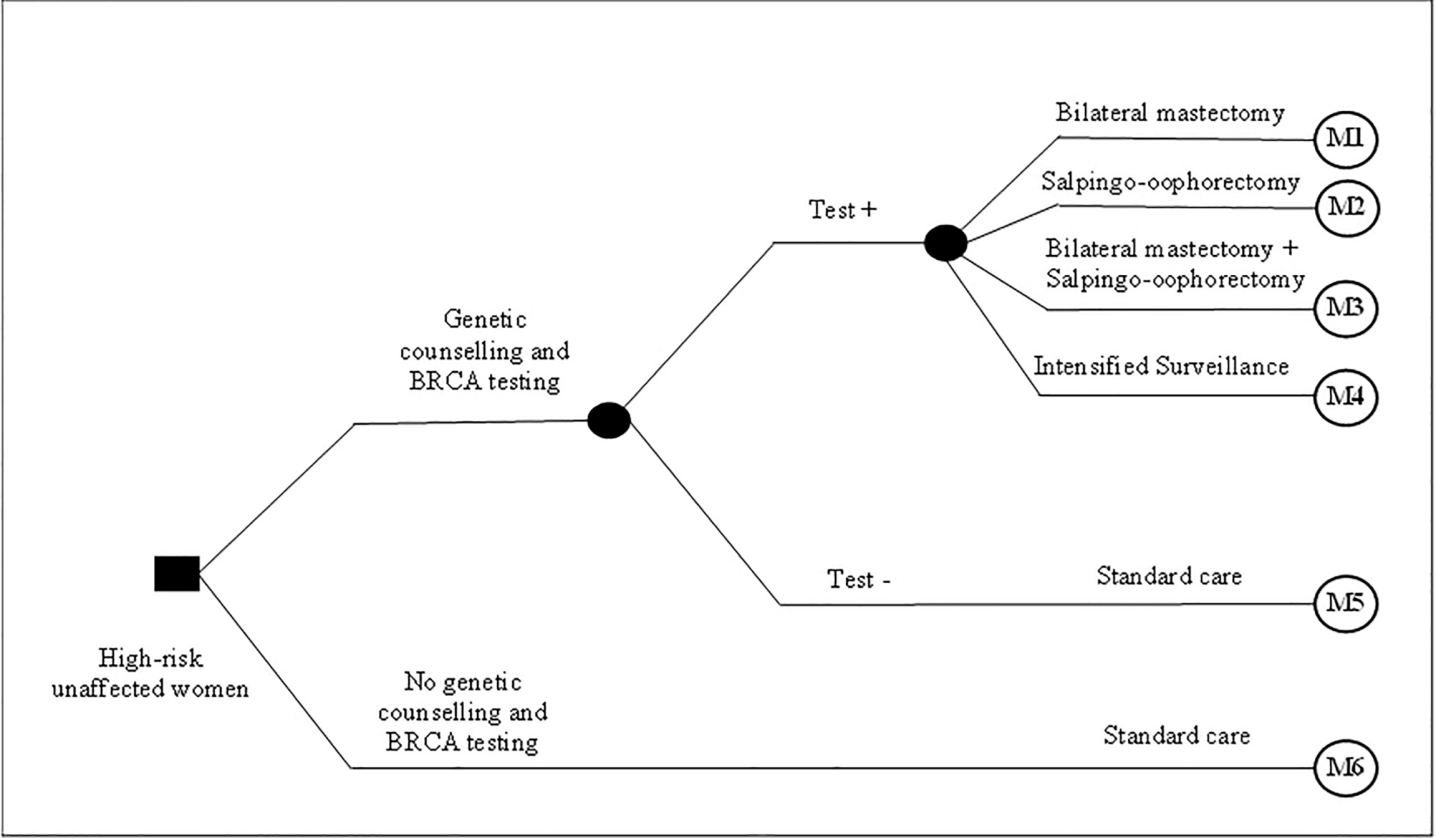
Decision model presenting compared strategies and high-risk reduction options.

The compared strategies consisted of carrying out the genetic counselling and *BRCA*1/2 genetic testing followed by different surgical/non-surgical preventive options, compared to not performing genetic testing and carrying out these preventive/risk reduction measures. For this model, we considered the clinical criteria for offering genetic testing (and preventive options for carriers) recommended by the National Comprehensive Cancer Network (NCCN) guideline (8). Women identified as *BRCA1/2* mutation carriers were offered four alternatives based on clinical/genetic criteria and personal choice: (i) intensified surveillance with MRI and bilateral mammography annually and breast specialist consultation, CA 125 exam, and transvaginal ultrasonography biannually; (ii) salpingo-oophorectomy; (iii) bilateral mastectomy; or (iv) both salpingo-oophorectomy and bilateral mastectomy (2, 8–11). Women who tested negative and women in the control group (women who did not have genetic counselling and *BRCA* testing) were treated in consonance with the SUS standard care according to their age (e.g., bilateral mammography and medical consultation annually for HBOC women aged 30 years). **Figure 1** presents the compared strategies and the preventive options for high-risk women.

### Model Overview

**Figure 2:**
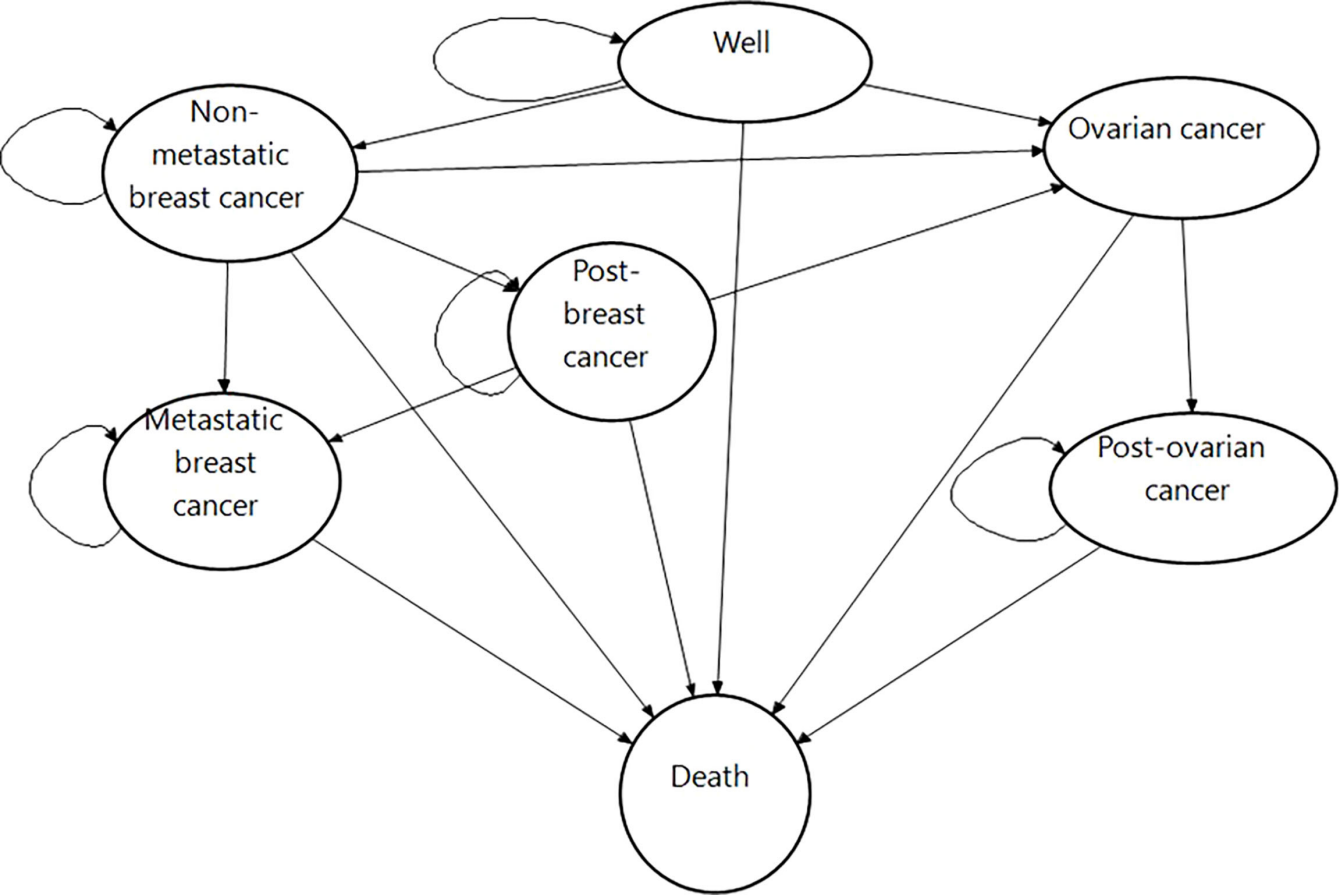
Markov diagram.

The Markov model structure comprises the states “well,” “non-metastatic breast cancer,” “metastatic breast cancer,” “ovarian cancer,” “post breast cancer,” “post ovarian cancer,” and “death” (absorbing state) (**Figure 2**).

Women in the model started in the state well and could go to the states breast or ovarian cancer or die. From breast cancer, they could either stay there, go to post breast cancer, or develop metastatic breast cancer, or ovarian cancer. Women with contralateral breast cancer returned to the initial breast cancer state. The transition from ovarian to breast cancer was not included due to the low incidence of ovarian cancer and its high mortality rates (1).

These states reflect possible clinical events for high-risk women for HBOC. The well state comprises women not diagnosed with cancer; the non-metastatic breast cancer state includes women in the first year after the diagnosis of first or contralateral breast cancer. The metastatic breast cancer state comprehends the first year of diagnosis of disseminated neoplastic cells in an organ distinct from the breast. Likewise, the ovarian cancer state includes women in the first year of diagnosis. Post-cancer states were modeled using tunnel states to reflect annual follow-up costs, utilities, and probabilities after cancer diagnosis until year 5. From the sixth year on, the patients stay in the post-cancer state unless other events occur. The ovarian cancer state was not separated between non-metastatic and metastatic due to its high risk of mortality which is caused by the difficulty to obtain an early detection of the disease (23).

### Probabilities

Transition probabilities were obtained mainly from the Barretos Cancer Hospital (BCH) dataset (**Table 1**). The BCH is a Brazilian philanthropic health institution specialized in cancer care, from prevention to treatment. It is a cancer center (non-profit foundation) that offers services through the Brazilian SUS. However, it differs from other public hospitals because it can receive donations from society, auctions, or organizations. Currently, the *BRCA* genetic test is not offered by hospitals operating in the Brazilian public health system, but the BCH can provide the test for its patients due to funds from donations obtained (36).

**Table 1:** Input data on annual probabilities and utilities and their sources.

Variable	Value (SD)	Sources
Probabilities		
To be tested positive with a genetic test	0.18	(BCH)
Choice of prophylactic option		
Mastectomy	0.03 (0.02–0.04)	(BCH)
Oophorectomy	0.12 (0.10–0.14)	(BCH)
Both	0.12 (0.09–0.17)	(BCH)
From well to BC		
Carriers	30–34 y.o.: 0.012 (0.01–0.013), 35–39: 0.016 (0.014–0.017), 40–44: 0.022 (0.020–0.024), 45–49: 0.027 (0.025–0.029), 50–54: 0.029 (0.027–0.031), ≥ 55 0.037 (0.033–0.040)	(BCH)
Non-carriers	30–34: 0.0011 (0.0001), 35–39: 0.0017 (0.0001), 40–44: 0.002 (0.0002), 45–49: 0.004 (0.0004), 50–54: 0.006 (0.0006), ≥ 55: 0.008 (0.0008)	(24, 25)
From well to OC		
Carriers	0.013 (0.052)	(BCH)
Non-carriers	0.00008728	(26, 27)
From well to death	30–34: 0.004, 35–39: 0.006, 40–44:0.009, 45–49: 0.013, 50–54: 0.019, 55–59: 0.028, 60–64: 0.043, 65–69: 0.065, 70–74: 0.1, 75–79: 0.16, 80–84: 0.25, 85> 1	(28)
From BC or post-BC to BC		
Carriers	0.069 (0.054–0.091)	BCH
Non-carriers	0.003 (0.001)	(29)
From BC or post-BC to death (BC mortality)	0.006 (0.004)	(BCH)
From BC or post-BC to Metastatic BC	0.0134 (0.0097–0.01737)	(BCH)
From BC or post-BC to OC	0.007 (0.004–0.010)	(BCH)
From metastatic BC to death	y1: 0.37 (0.31–0.48), y2: 0.61 (0.53–0.73), y3: 0.76 (0.68–0.86), y4: 0.85 (0.78–0.92), y5: 0.9 (0.85–0.96)	(BCH)
From OC to death	y1: 0.10 (0.04), y2: 0.18 (0.06); y3: 0.25 (0.07), y4: 0.32 (0.08), y5: 0.39 (0.08)	(BCH)
Development of breast cancer		
Women with bilateral mastectomy	There are no cases reported in BCH	(BCH)
Women with oophorectomy ^b^	30–34: 0.014 (0.01–0.013), 35–39: 0.016 (0.014–0.017), 40–44: 0.022 (0.020–0.024), 45–49: 0.027 (0.025–0.029), 50–54: 0.029 (0.027–0.031), ≥ 55 0.037 (0.033–0.040)	(BCH)
Women with bilateral mastectomy and oophorectomy	There are no cases reported at BCH	(BCH)
Development of ovarian cancer		
Women with bilateral mastectomy	There are no cases reported at BCH	(BCH)
Women with oophorectomy	0.01 (0.0004–0.32)	(BCH)
Women with bilateral mastectomy and oophorectomy	0	(BCH)
Utility values		
Well, at age 30	0. 920 (0.0072)—baseline	(30)
Annual decrease due to age	0.00029	(30)
Healthy high-risk women	Multiplier: 0.92	(31)
Prophylactic mastectomy, oophorectomy or both	Multipliers: 0.88 (0.22), 0.95 (0.1), 0.83 (0.1)	(9)
Annual increase after prophylactic mastectomy or both oophorectomy and mastectomy in years 2–5	0.008 (0.001), 0.02 (0.011)	Assumption based on previous modeling studies (22, 32)
BC	Multiplier: 0.77 (0.18)	(33)
Post-BC	Multiplier 0.79 (0.18)	(33)
Annual increase after BC in years 2–5	0.0021 (0.0007)	Assumption based on previous modeling studies (22, 32)
Metastatic BC	Multiplier: 0.64 (0.12)	(34).
OC	Multiplier: 0.34 (0.30)	(35)
Post-OC	Multiplier: 0.83 (0.25)	(35)
Annual increase after OC in years 2–5	0.111 (0.022)	Assumption based on previous modeling studies (22, 32)
		

BC, breast cancer; OC, ovarian cancer; SD, standard deviation; BCH, Barretos Cancer Hospital.

b It was assumed that it has the similar breast cancer risk of BRCA carrier women.

The probabilities that could not be retrieved from the BCH dataset were taken preferably from sources that reflected the Brazilian population, e.g., the National Cancer Institute of Brazil (INCA), Brazilian Geography and Statistics Institute (IBGE), and the WHO. The specific references are provided in **Table 1**.

In the BHC dataset, data were available for 2,307 women who performed BRCA1/2 genetic testing from 2011 to 2020. There were 1,544 index cases (i.e., the first member of the family to be tested)— among which there were 275 carriers and 1,269 non-carriers—and 763 first- or second-degree relatives (i.e., 356 carriers and 407 non-carriers). Using Kaplan–Meier in the SPSS software, the transition probabilities were calculated. We defined a different group eligible at baseline for each of the transition probabilities calculated to avoid selection bias regarding a previous cancer diagnosis. Briefly, we considered data from first- or second-degree relatives identified to be carriers to calculate the probability of opting for a prophylactic surgery and the respective risk of developing breast or ovarian cancer afterward. Then, we considered data from index cases to calculate transition probabilities for *BRCA* carriers from the state “well” to “cancer” only for those not submitted to prophylactic surgeries.

As BCH is a reference cancer center for women at high risk, women who tested negative were not followed up at BCH but referred back to the system for general population screening according to their ages, considering that women who tested negative have been shown to have the same risk as the general population (13–16). Thus, the incidence of cancer and mortality for women tested negative were taken from Brazilian registries (26, 27).

Because data on the probability of BC recurrence among *BRCA* non-carriers in Brazil are not available, we used the cumulative 10-year risk of secondary contralateral breast cancer for German non-carriers (29). The data choice was based on similarities in definitions of health states (37).

### Utility Data

Utility data were extracted from published studies from a systematic literature search in the PubMed database (**Supplementary Table 1**). When possible, studies reporting utility values for the Brazilian population were preferred. Due to methodological heterogeneity among the studies reporting utilities (37), we used relative utility values applying decrements to the baseline (“well”) state (30).

In our study, due to the distress of knowing to have a mutation and distress caused by undergoing risk-reducing surgery, utilities decreased for high-risk women (31), risk-reducing surgeries, and breast or ovarian cancer (9). All women that entered the model were considered high-risk for HBOC. Thus, if they have a negative test result, it was assumed that their utility increases to the utility of healthy women, obtained from Sullivan et al. (2005).

The decrements in utilities for high-risk women for HBOC were based on EQ-5D values of women in Croatia (31). Utilities for the prophylactic surgeries were obtained from Grann et al. (2010), in which a time trade-off instrument (TTO) was applied to *BRCA*-mutated Canadian women. Decreased utilities following prophylactic mastectomy and prophylactic salpingo-oophorectomy were assumed to increase linearly within 5 years to regain the age-specific utility of a high-risk woman, as suggested by other modeling studies (22, 32).

Utilities for breast cancer and post-breast cancer were based on the EQ-5D values from a prospective cohort of Brazilian women newly diagnosed with breast cancer and treatment naïve ^32^. The utilities for metastatic breast cancer were extracted from a meta-regression of studies using a Standard Gamble approach (34).

Weighted average utilities for ovarian cancer and post-ovarian cancer were obtained from (35), in which utilities were measured in populations from different countries using the Standard Gamble approach. Following assumptions of other modeling studies (22, 32), it was assumed that women’s utility declines as a result of breast or ovarian cancer and then increases linearly for 5 years to reach the age-specific utility of a post-cancer state. **Table 1** presents all input data regarding probabilities and utilities and their sources.

### Cost Data

Adopting the perspective of the SUS, direct medical annual costs were calculated for each Markov model health state. Cost data were expressed in Brazilian currency (Reais). The unit cost values for 2021 were obtained from the official SUS database, namely, the Table of Procedures, Medications and Ortheses, Prostheses, and Special Materials for the National Health System (DATASUS Tabnet). Resource use (e.g., diagnostic exam and clinical procedures) was estimated based on recommendations from the NCCN guidelines and interviews with one oncologist and one gynecologist. The cost of breast cancer treatment was calculated as a weighted average that considered cancer molecular type and stage at BCH cohort, indicating a higher proportion of breast cancer diagnosis in the early stage for first- or second-degree relative women than index women (**Supplementary Table 2**).

Considering that the cost of treatment is potentially lower for breast cancer diagnosed at early stages, the annual mean cost was calculated for these two subgroups from the BCH dataset: (i) index women, that is, *BRCA*-mutated women who had breast cancer before the genetic test, and (ii) first- or second-degree relative women, that is, the *BRCA*-mutated women who had cancer after the test.

The cost of the genetic test refers to the price paid by the BCH (**Table 2**) and was obtained from the Laboratory of Molecular Diagnostics from BCH, considering reagents and personal and taking into consideration the costs of performing BRCA1/BRCA2 analysis by next-generation sequencing (NGS) complemented by rearrangement analysis by multiplex length polymorphism analysis (MLPA). Besides, the genetic test cost was calculated as the mean cost of one index and two relative women tested. The costs of intensive screening included the provision of magnetic resonance and bilateral mammography once a year, breast specialist consultation, CA 125 exam, and transvaginal ultrasound twice per year. The costs concerning standard care were related to bilateral mammography and breast specialist consultation once a year. **Table 2** summarizes the cost input data used in the sensitivity analysis. To facilitate comparisons with costs from other countries, conversion of the results presented in Brazilian real (R$) to United States dollar ($) was performed by using a web-based tool (CCEMG—EPPI-Centre Cost Converter). This tool considers the Gross Domestic Product deflator index and the Purchasing Power Parities for GDP (“PPP values”) to convert currencies.

**Table 2:** Costs^a^ of breast and ovarian cancer (R$ and US$).

Costs of test, preventive surgeries and surveillance, value in R$ (US$)					
*BRCA* testing		1135^b^ (524.98)			
Intensive screening and genetic counseling		428.85 (198.36)			
Standard care		55.00 (25.44)			
Prophylactic mastectomy		3484.26 (1611.59)			
Prophylactic salpingo-oophorectomy		621.00 (287.23)			
Both prophylactic mastectomy and salpingo-oophorectomy		4105.26 (1898.83)			
**Breast cancer treatment**					
**Breast cancer—index**					
Cost per procedure group (%)	1° year	2° year	3° year	4° year	5° year
Diagnostic (%)	1,085.93 (6.96)	317.62(33.12)	279.03 (38.80)	279.03 (38.80)	279.03 (38.80)
Surgical procedures (%)	2,219.87 (12.46)	-	-	-	-
Clinical procedures					
Hormonotherapy (%)	440.00 (2.47)	440.00 (45.88)	440.00 (61.19)	440.00 (61.19)	440.00 (61.19)
Neoadjuvant chemotherapy (%)	5,489.87 (30.81)	-	-	-	-
Adjuvant chemotherapy (%)	2,673.44 (15.00)	201.33 (20.99)			
Radiotherapy (%)	5,904.00 (33.14)	-	-	-	-
Sum per health state per year	17,813.11	958.95	719.03	719.03	719.03
**Breast cancer—first- or second-degree relatives**					
Cost per procedure group (%)	1° year	2° year	3° year	4° year	5° year
Diagnostic (%)	1021.89 (6.17)	317.38 (33.97)	279.03 (38.80)	279.03 (38.80)	279.03 (38.80)
Surgical procedures (%)	2237.39 (13.52)	-	-	-	-
Clinical procedures (%)					
Hormonotherapy (%)	440.00 (2.66)	440.00 (47.09)	440.00 (61.19)	440.00 (61.19)	440.00 (61.19)
Neoadjuvant chemotherapy (%)	4,455.75 (26.93)	-	-	-	-
Adjuvant chemotherapy (%)	2,481.06 (15.00)	176.89 (18.9)			
Radiotherapy (%)	5,904.00 (35.69)	-	-	-	-
Sum per health state per year	16,540.09	934.27	719.03	719.03	719.03
**Metastatic breast cancer**					
Cost per procedure group (%)	1° year	2° year	3° year	4° year	5° year
Diagnostic (%)	3,124.01 (16.98)	2,956.77 (17.10)	1,690.37 (11.30)	1699.77 (10.68)	1,699.77 (10.68)
Clinical procedures (%)					
Hormonotherapy (%)	440.00 (2.39)	1,663.45 (9.62)	138.62 (0.92)	-	-
Palliative chemotherapy (%)	12,340.32 (67.09)	12,665.36 (73.27)	13,120.92 (87.76)	14,214.25 (89.31)	14,214.25 (89.31)
Radiotherapy (%)	2,488.28 (13.52)	-	-	-	-
Sum per health state per year	18,392.61	17,285.58	14,949.91	15,914.02	15,914,02
**Ovarian cancer**					
Cost per procedure group (%)	1° year	2° year	3° year	4° year	5° year
Diagnostic (%)	544.18 (3.83)	270.23 (3.15)	259.77 (4.0)	240.68 (100)	240.68 (100)
Surgical procedures (%)	829.10 (5.83)	-	-	-	-
Clinical procedures (%)					
Chemotherapy (%)	6,624.18 (46.57)	-	-	-	-
Palliative chemotherapy (%)	6,227.46 (43.78)	8,303.28 (96.85)	6,227.46 (96.0)	-	
Sum per health state per year	14,224.92	1,654.11	6,487.23	240.68	240.68

a Considering high uncertainty in cost values, for sensitivity analysis, an assumption of 40% standard deviation was made.

b Mean cost considering one index and two relative women tested.

### Model Validation and Sensitivity Analyses

To validate the model, we consulted experts on the adequacy of input data and the conceptual appropriateness of the model. Technical accuracy was checked regarding data entry and potential programming errors (computerized model validation). For cross-model validation, we assessed the extent to which other models for breast cancer prevention came to different conclusions (38). We performed deterministic sensitivity analyses by varying probabilities and utilities considering uncertainty within the respective ranges or confidence limits to characterize overall uncertainty in the outcome measures. To obtain a comprehensive range, the costs were varied within the 40% range, as suggested by (32). Besides, a probabilistic sensitivity analysis with Monte Carlo simulation (10,000 interactions) was conducted. Gamma distributions were used for cost parameters. Probabilities and utilities were considered to be beta-distributed.

## Results

### Base-Case Analysis

The genetic counseling and *BRCA* testing strategy cost R$ 5,298 (U$ 2,450.51) in the base-case scenario, resulting in an incremental cost of R$ 1,796 (U$ 830.71) compared with the non-testing strategy. Accordingly, women offered the genetic test had an incremental gain of 0.2 QALYs and 0.2 LYG. The incremental cost-effectiveness ratio (ICER) for the base-case analysis was R$ 11,900.31 (U$ 5,504.31) per QALY and 10,988.67 (U$ 5082.64) per LYG.

In the Brazilian scenario, an exact value of the cost-effectiveness threshold to be applied by the National Commission for the Incorporation of Technologies (CONITEC) in the SUS was not defined (39). However, based on values of thresholds presented in CONITEC recommendation reports, the study by (40) suggested a three-level threshold: low (<R$ 25,000), medium (R$ 25,000 to R$ 70,000), and high (>R$ 70,000). Therefore, to be more conservative, the present study considered a willingness to pay of R$ 25,000. Base-case results are described in **Table 3** and **Supplementary Figure 1**.

**Table 3:** Base case results.

Strategy	Cost (R$)	Incremental costs (R$)	QALYs	Incremental QALYs	LYG	Incremental LYG	ICER (R$)	
							Costs/QALY	Costs/LYG
**No testing**	3,502		14.4		16,0			
**Testing**	5,298	1,726	14.6	0.2	16,01	0.2	11,900.31	10, 988.67

### Sensitivity Analyses

In the deterministic sensitivity analysis, the variables with the largest impact on the ICER were the discount rate, probability of moving from well to breast cancer after both risk-reducing surgeries, cost of the genetic test, probability from well to breast cancer after salpingo-oophorectomy, and breast cancer treatment costs. A discount rate of zero would reduce the ICER to R$ 3,336.10/QALY (U$ 1,543.06/QALY), and a discount rate of 10% would increase the ICER to R$ 31,617.71/QALY (U$ 14,624.29/QALY). In a scenario where the probability of moving from well to breast cancer after both risk-reducing surgeries is higher (0.08), the ICER increases to R$ 36,362.66/QALY (U$ 16,818.99/QALY). Moreover, in the scenario that only one first- or second-degree relative woman could be tested per index woman tested instead of two, the costs of a genetic test would increase to R$ 2,035.00 (U$941.26/QALY), increasing the ICER to R$ 17,862.54/QALY (U$ 8262.04/QALY), whereas, if the costs of a genetic test per woman decreased to R$ 685 (U$ 316.84) due to testing four relatives per index woman, the ICER would decrease to R$ 8,919.19/QALY (U$ 4125.43/QALY).

The cost-effectiveness ratio was also sensitive to the probability of moving from well to ovarian cancer after salpingo-oophorectomy; if this probability was higher (0.03), the ICER would increase to R$ 16,766.88, while if it was smaller (0.004), the ICER would decrease to R$ 10,179.45. Regarding the breast cancer treatment cost for non-tested women, assuming a 40% higher cost of breast cancer treatment for the non-testing group, the ICER would decrease to R$ 10,630.77/QALY (U$ 4,917.10/QALY). However, if the breast cancer treatment for this group was 40% lower, the ICER would increase to R$ 17,813.11/QALY (U$ 8,239.18/QALY).

**Figure 3:**
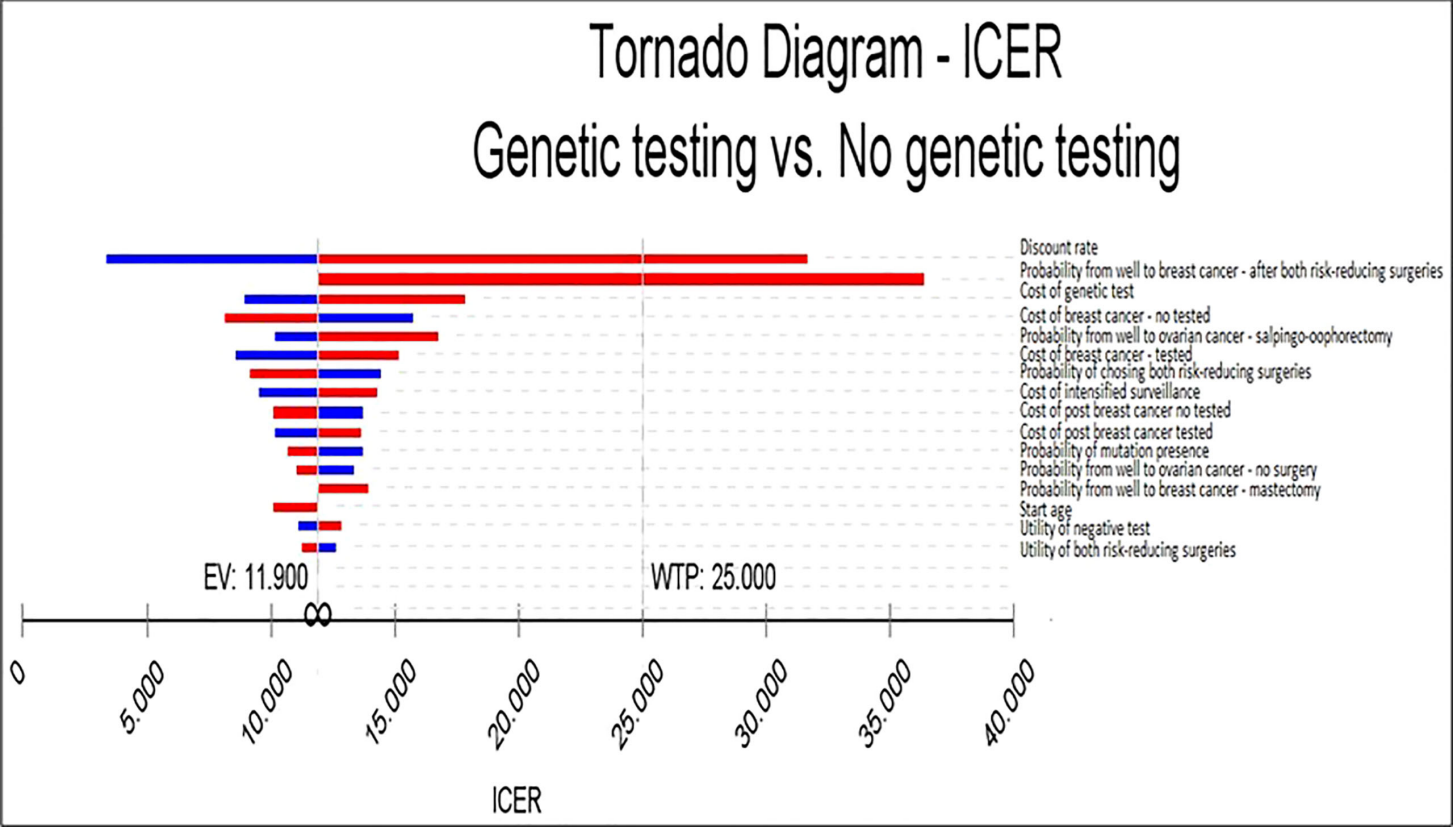
Deterministic sensitivity analyses.

The tornado diagram indicates that for almost all intervals considered in the analysis, the testing strategy is considered cost-effective when compared to the no testing strategy (**Figure 3**), considering a willingness to pay R$ 25,000 (U$ 11,563.37) per QALY (**Supplementary Table 3**).

**Figure 4:**
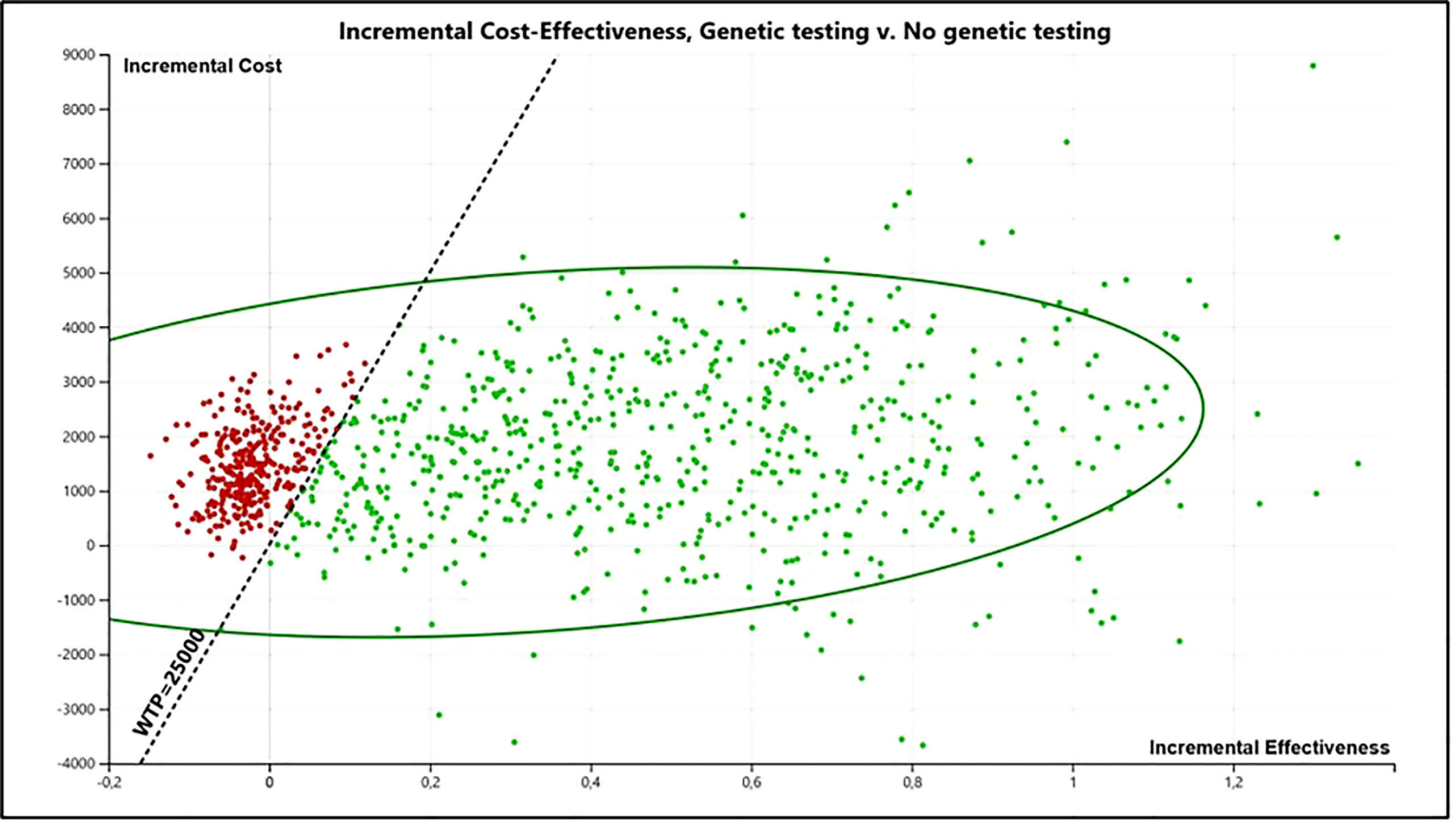
Incremental cost-effectiveness plane from the probabilistic sensitivity analyses (PSA) (10,000 interactions).

**Figure 4** presents the incremental cost-effectiveness plane from the probabilistic sensitivity analyses.

**Figure 5:**
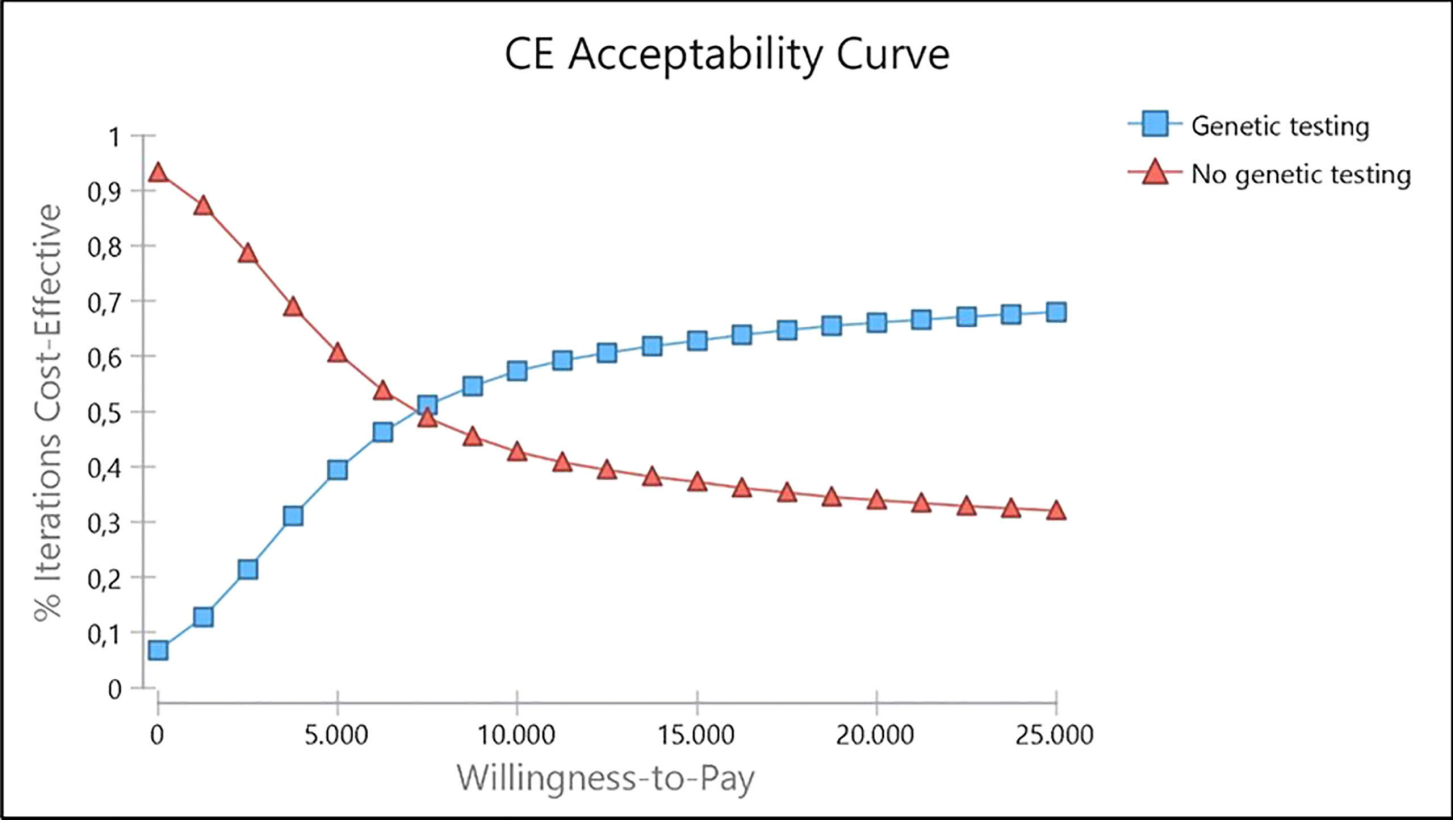
Cost-effectiveness acceptability curve for genetic testing strategy provided to Brazilian women.

The cost-effectiveness acceptability curve showed a probability of genetic testing being cost-effective of 68.03% at a willingness to pay (WTP) of R$ 25,000/QALY (U$ 11,563.37/QALY) (**Figure 5**). Besides, it becomes cost-effective at a minimum threshold of R$ 7,500/QALY (U$ 3,469.01/QALY). **Supplementary Figure 2** presents the incremental net monetary benefit (INMB) versus willingness to pay analysis.

While perceived by the upper-middle-income countries’ government as a potentially highly costly intervention, as our results show, carrying out the genetic counseling, *BRCA*1/2 genetic tests, and preventive options in women at high risk for HBOC is a very cost-effective intervention compared to not carrying out these actions when considering a willingness to pay of R$ 25,000/QALY. The ICER for the base-case analysis was R$ 11,900.31 (U$ 5,504.31). The sensitivity analysis also revealed a superiority of the testing strategy. The tornado diagram points out that genetic testing is cost-effective for all scenarios. The probabilistic sensitivity analysis indicates a probability of genetic testing being cost-effective of 68.03%.

The main novelty of our results is that this is the first study for upper-middle-income countries on BRCA genetic tests whose probabilities were mainly extracted from trial-based analysis with Brazilian registries (i.e., penetrance of *BRCA*, rates of uptake prophylactic procedures, breast or ovarian cancer development, etc.). It shows that genetic testing can be cost-effective even in upper-middle-income countries. The use of patients’ clinical data increases the representativeness of the results of our analysis for Brazilian women. This real-world evidence provides a more accurate representation of the target population for several reasons. First, the penetrance of BRCA is highly associated with the genetic profile of the population, and the Latin-American population is underrepresented in most international registries and databases. Second, rates of uptake prophylactic procedures vary widely worldwide since these are highly preference-sensitive decisions influenced by sociocultural factors. Therefore, our results add to the existing literature by demonstrating the cost-effectiveness of BRCA in a model that accurately reflects the epidemiology and the preferences of Latin-American women (from Brazil) at-risk for hereditary breast cancer.

Previous Markov model studies evaluated the cost-effectiveness of genetic *BRCA* testing for breast or ovarian cancer in high-risk women compared to no test in Brazil (17, 22, 41). All of them concluded that *BRCA* testing seems to be the cost-effective strategy with ICERs of R$ 24.264/QALY (22), R$ 908,52 per case of cancer avoided (41), and $ 20,995/QALY (17).

The Brazilian study by Simoes Correa-Galendi et al. (2021) had a similar structure. However, the data were extracted exclusively from the literature. For instance, the uptake rates taken from a UK cohort were 0.09 for prophylactic mastectomy and 0.22 for prophylactic salpingo-oophorectomy. In contrast, the uptake rates identified in our study were 0.03 and 0.12, respectively. These data demonstrate huge differences when considering real-world data from the Brazilian population. According to a recent systematic review by (42), the variability of uptake rates of the risk-reducing surgeries might be explained by several factors, such as (i) cultural differences, (ii) individual-related factors, (iii) age-dependent factors, and (iv) an improved acceptance of preventive surgeries over time. Besides these reasons, it is worth mentioning that economic factors might have also influenced the Brazilian uptake rates identified in our study, for instance, the lack of access to risk-reducing surgeries in the public setting and patients’ expenses with transport, accommodation, and absence from work, once the services that provide these surgeries are usually in cancer reference hospitals or large capitals.

Ramos et al. (2019) evaluated the preventive strategies only for the relatives of patients diagnosed with ovarian cancer, while the present study analyzed preventive strategies for first- or second-degree relatives of patients diagnosed with breast or ovarian cancer. Finally, the study by (17) differs from the present research because they evaluate the population-based BRCA testing, which possibly is why the ICER is higher.

The clinical data used in the present study indicate a probability of 82% to receive a negative *BRCA* genetic test. Considering that breast or ovarian cancer risk among non-carrier women from positive families is similar to the general population (13–16), it is important to note that there is still an around 50% probability that the relative will get a negative test and no longer be considered at high risk.

In this context, the benefit that a negative test can bring to patients is not trivial and should be considered in economic modeling studies (43). Our study considers that by obtaining a negative result, the unaffected patient (from a BRCA-mutated family) stops having the utility of a patient at high risk for HBOC and starts obtaining the utility of a woman without high risk at her age due to the reduction in their level of distress (44). This increase in utility occurs as the woman may no longer be excessively concerned with intensified surveillance and breast and/or ovarian cancer (31, 45). A small utility increase due to the relief of receiving a negative test result was also assumed by a previous modeling study (46). However, while Holland et al. justified this increase in utility due to an assumption, in the present study, this analysis is supported by recent evidence from (31).

Of note, our present study provided a conservative analysis considering that only two female relatives are tested for each index. The cost of testing an index patient in Brazil is around R$ 1,800 (U$ 832—including labor, reagents, and rearrangement analysis), while the cost of testing a relative of the patient is R$ 235 (U$ 108). Thus, the average unit cost of testing a family member is calculated at R$ 1135 (U$ 525). However, if we consider that it is possible to test a higher number of family members for each index tested, the unit cost of testing a family member would be reduced, making the genetic testing strategy even more cost-effective, as its ICER would be reduced.

The main strength of the present study was to conduct a trial-based analysis to obtain transition probabilities from the data of women tested from 2011 to 2020 in a Brazilian hospital. Another advantage was the cost data analysis. The unit cost values were obtained from the official Brazilian Health System database. Concerning breast cancer treatment, the costs were calculated separately for molecular types (triple-negative, Luminal A, Luminal B, and Her2+) and stages (47, 48) to reflect a realistic scenario of the resource use. In consonance with growing evidence (17, 32, 49), the present study also highlights the genetic testing contribution to earlier cancer detection. For instance, our cohort showed a high rate of stage III/IV in the index patients (52.08%), and a tendency of earlier diagnosis (stages I/II) in patients who performed genetic testing before a cancer diagnosis (58.67%). Besides, even though the uptake of risk-reducing surgeries was low in our cohort, these patients had access to intensified surveillance with breast magnetic resonance imaging (MRI) which might also contribute to earlier diagnosis.

Limitations might have affected the results. Although data from BCH were preferred, probabilities of events that happen with *BRCA* non-carriers had to be taken from the literature because those patients were not followed up at the BCH. In addition, because of the sample size of the BCH database, the transition probabilities calculated in the present study might not generalize to the entire Brazilian population. Another limitation of the model is the unknown risk of *BRCA*-negative women with a family history of cancer compared with cancer risks observed in the general population. It was considered that non-carriers of genetic mutations that came from positive families did not show an increased risk for HBOC (13–16) and, according to the NCCN guideline, should have a standard care (8). Lastly, because most data on utility specific to the Brazilian population are not available, we used studies from other countries that reported the most similar health-related quality of life; moreover, the available data on utility are not homogeneous. Nevertheless, the sensitivity analysis reveals that for all intervals considered, the testing strategy is considered cost-effective compared to the no testing strategy, considering a willingness to pay of R$ 25,000 per QALY.

Importantly, the present results can support policy development on the topic. Currently, genetic testing is not covered by the Universal Health Coverage in Brazil. The present study uses Brazilian women’s clinical data to support the argumentation that the Brazilian public health system should offer the *BRCA* genetic test for women with a family history that leads to increased risk for HBOC. Our results indicate that a comprehensive genetic test-and-screen strategy for high-risk Brazilian women results in a substantial gain of QALY at moderate additional costs. Although genetic testing followed by preventive surgeries appears to be the most economically advantageous option, women’s preferences should always be considered and drive the final treatment decision.

## Conclusion

In this study, we showed that a screen-and-treat strategy for healthy women at risk for HBOC results in more QALYs and moderately more costs, with an ICER of R$ 11,900.31 (U$ 5,504.31) per QALY gained. The cost-effectiveness of the screen-and-treat intervention depends on a still undecided cost-effectiveness threshold for Brazil, but it would be cost-effective considering a willingness to pay of R$ 25,000 (U$ 11,563.37) per QALY. These results might be reproducible in other upper middle-income countries.

## Data Availability Statement

The original contributions presented in the study are included in the article/**Supplementary Material**. Further inquiries can be directed to the corresponding author.

## Ethics Statement

The studies involving human participants were reviewed and approved by Barretos Cancer Hospital’s research ethical committee (approval number: 56164716.9.0000.5437). Written informed consent for participation was not required for this study in accordance with the national legislation and the institutional requirements.

## Author Contributions

Conceptualization, ML and EP. Methodology, ML, JC-G, AA, OM and EP. Software, ML and JC-G. Validation, ML, JC-G and OM. Formal analysis, ML. Investigation, ML and HG. Resources, HG and EP. Data curation, ML. Writing—original draft preparation, ML and RG. Writing—review and editing, EP, JO, JC-G, OM, RR, AC and EM. Visualization, ML. Supervision, EP. Project administration, EP. Funding acquisition, EP. All authors have read and agreed to the published version of the manuscript.

## Funding

This project was funded through grants from the National Oncology Care Support Program (PRONON, Grant number 25000.056766/2015-64) from the Brazilian Ministry of Health. EIP and RMR are recipients of the National Council for Scientific and Technological Development (CNPq) productivity fellowships. The study sponsors had no involvement in the study design, collection, analyses or interpretation of data.

## Conflict of Interest

The authors declare that the research was conducted in the absence of any commercial or financial relationships that could be construed as a potential conflict of interest.

The reviewer MD declared a shared affiliation, with no collaboration, with the authors ML and JO to the handling editor at the time of review.

## Publisher’s Note

All claims expressed in this article are solely those of the authors and do not necessarily represent those of their affiliated organizations, or those of the publisher, the editors and the reviewers. Any product that may be evaluated in this article, or claim that may be made by its manufacturer, is not guaranteed or endorsed by the publisher.
