# Supplementary material for: Cost-Effectiveness of BRCA 1/2 Genetic Test and Preventive Strategies: Using Real-World Data From an Upper-Middle Income Country
Source: Front Oncol. 2022 Jul 11;12:951310. doi: 10.3389/fonc.2022.951310 (PMC9309566; doi:10.3389/fonc.2022.951310)
Supplement: Supplementary file 1 [file DataSheet_1.docx]

Supplementary material

# Supplementary data

**Supplementary Table 1 |** Utilities Search strategies

**Supplementary Table 2 |** Cancer molecular type and stage at BCH cohort

**Supplementary Figure 1 |** Cost-effectiveness base case results

**Supplementary Figure 2** | INMB versus willingness to pay for both strategies

**Supplementary Table 3 |** Tornado text report

Supplementary Table 1 | Utilities Search strategies

| Breast Neoplasm [Mesh] | Breast Neoplasm OR Neoplasm, Breast OR Breast Tumors OR Breast Tumor OR Tumor, Breast OR Tumors, Breast OR Neoplasms, Breast OR Breast Cancer OR Cancer, Breast OR Mammary Cancer OR Cancer, Mammary OR Cancers, Mammary OR Mammary Cancers OR Malignant Neoplasm of Breast OR Breast Malignant Neoplasm OR Breast Malignant Neoplasms OR Malignant Tumor of Breast OR Breast Malignant Tumor OR Breast Malignant Tumors OR Cancer of Breast OR Cancer of the Breast OR Mammary Carcinoma, Human OR Carcinoma, Human Mammary OR Carcinomas, Human Mammary OR Human Mammary Carcinomas OR Mammary Carcinomas, Human OR Human Mammary Carcinoma OR Mammary Neoplasms, Human OR Human Mammary Neoplasm OR Human Mammary Neoplasms OR Neoplasm, Human Mammary OR Neoplasms, Human Mammary OR Mammary Neoplasm, Human OR Breast Carcinoma OR Breast Carcinomas OR Carcinoma, Breast OR Carcinomas, Breast | [#1] 463,422 |
| --- | --- | --- |
| Ovarian Neoplasms[Mesh] | Neoplasm, Ovarian OR Ovarian Neoplasm OR Ovary Neoplasms OR Neoplasm, Ovary OR Neoplasms, Ovary OR Ovary Neoplasm OR Neoplasms, Ovarian OR Ovary Cancer OR Cancer, Ovary OR Cancers, Ovary OR Ovary Cancers OR Ovarian Cancer OR Cancer, Ovarian OR Cancers, Ovarian OR Ovarian Cancers OR Cancer of Ovary OR Cancer of the Ovary | [#2]135,807 |
| quality-adjusted life years [Mesh] | Life Year, Quality-Adjusted OR Life Years, Quality-Adjusted OR Quality-Adjusted Life Year OR Year, Quality-Adjusted Life Years, Quality-Adjusted Life OR Quality Adjusted Life Years QALY OR Healthy Years Equivalents OR Equivalents, Healthy Years OR Healthy Years Equivalent OR Adjusted Life Years OR Adjusted Life Year OR Life Year, Adjusted OR Life Years, Adjusted OR Year, Adjusted Life OR Years, Adjusted Life | [#3]43,386 |
| quality of life[Mesh] | Life Quality OR Health-Related Quality Of Life OR Health Related Quality Of Life OR HRQOL | [#4]447,253 |
| [#1] OR [#2] |  | [#5] 571,805 |
| [#3] OR [#4] |  | [#6] 468,263 |
| [#5] AND [#6] |  | 17,416 |
|  | Limiting for Review | 6,336 |

**Supplementary Table 2** | Cancer molecular type and stage at BCH cohort

| Breast cancer molecular type proportions of *BRCA* mutated women | |
| --- | --- |
| Triple-negative | 48.66% |
| Luminal A | 3.83% |
| Luminal B | 42.15% |
| Her2+ | 5.36% |
| Percentage of BRCA1 and BRCA2 carriers | |
| BRCA 1 | 61.07% |
| BRCA 2 | 38.93% |
| Mean age of breast cancer diagnosis | 43.16 |
| Proportion of breast cancer stages at diagnosis for index women (i.e., tested after the cancer diagnosis) | |
| Stage I | 13.9% |
| Stage II | 34.89% |
| Stage III | 41.87% |
| Stage IV | 10.21% |
| Proportion of breast cancer stages at diagnosis for first and second-degree relative women (i.e., cancer diagnosis after testing) | |
| Stage I | 24.19% |
| Stage II | 35.48% |
| Stage III | 30.65% |
| Stage IV | 9.68% |

**Supplementary Figure 1** - Cost-effectiveness base case results


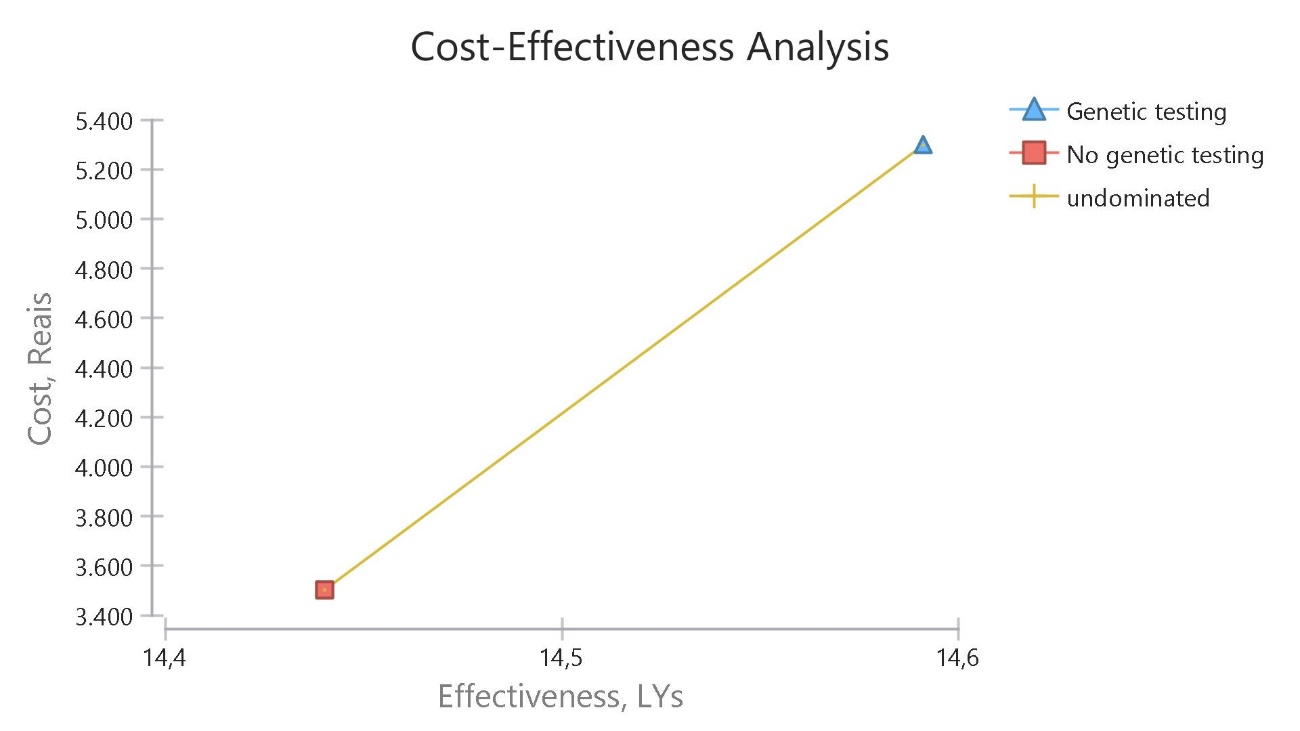


**Supplementary Figure 2** | INMB versus willingness to pay for both strategies


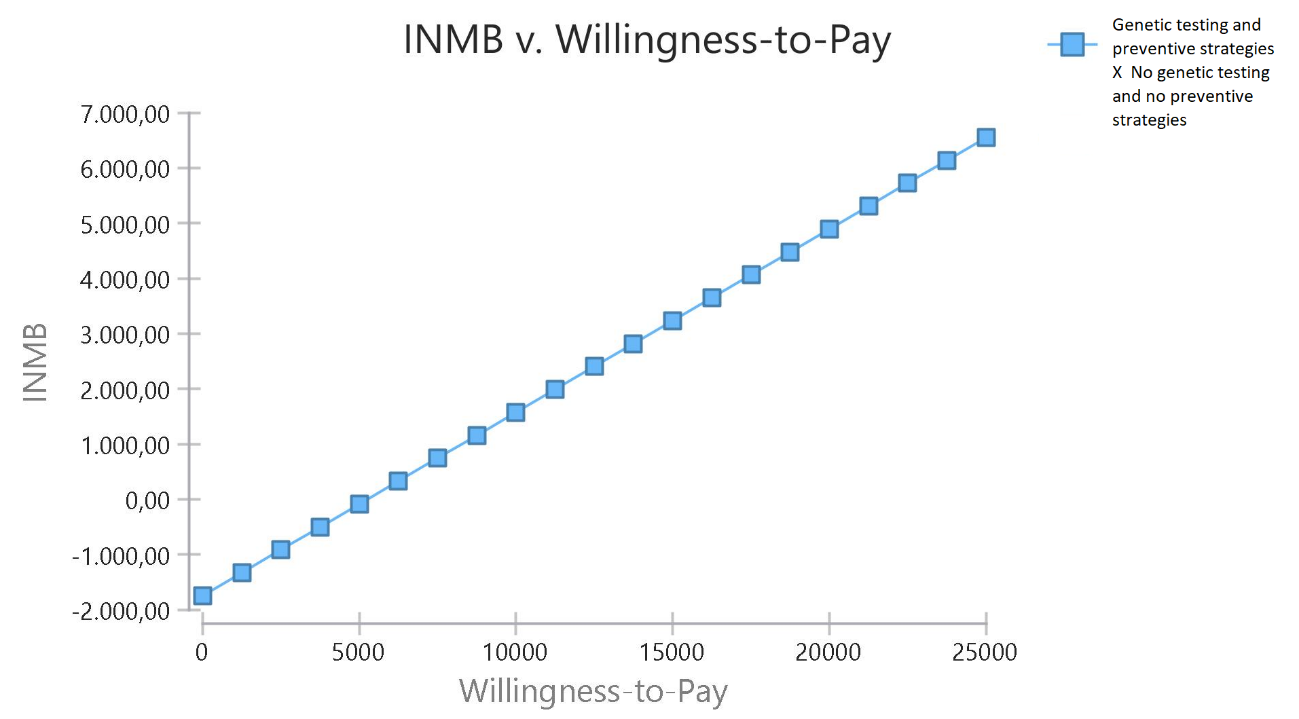


Supplementary Table 3 | Tornado text report

| Variable Name | Variable Low | Variable Base | Variable High | Impact | Low | High | Spread | Spread2 |
| --- | --- | --- | --- | --- | --- | --- | --- | --- |
| cDR | 0 | 0.05 | 0.1 | Increase | 333.610.577 | 3.161.771.245 | 2.828.160.668 | 79.984.927.655.077 |
| p_well_BC_both | 0 | 0 | 0.08 | Increase | 1.190.031.031 | 3.636.266.206 | 2.446.235.175 | 59.840.665.301.045 |
| c_genetic_test | 685 | 1135 | 2035 | Increase | 891.919.142 | 1.786.254.809 | 894.335.668 | 7.998.362.862.755 |
| c_breast_cancer_no_tested | 10630.77 | 17813.11 | 24805.14 | Decrease | 816.072.866 | 1.574.167.639 | 758.094.772 | 5.747.076.837.219 |
| p_well_OC_SOP | 0.00044 | 0.01 | 0.0329 | Increase | 1.017.945.248 | 1.676.688.383 | 658.743.134 | 4.339.425.171.921 |
| c_breast_cancer_tested | 9882.34 | 16540.09 | 23058.8 | Increase | 857.319.911 | 1.515.793.834 | 658.473.922 | 4.335.879.065.135 |
| p_both | 0.09 | 0.12 | 0.17 | Decrease | 918.950.894 | 1.440.132.889 | 521.181.995 | 2.716.306.722.984 |
| c_health_carriers_intensified_surveillance | 257.31 | 428.85 | 600.39 | Increase | 949.847.884 | 1.430.214.178 | 480.366.294 | 2.307.517.767.028 |
| c_post_breast_cancer_no_tested_valcol | 1 | 2 | 3 | Decrease | 1.006.556.063 | 1.373.505.998 | 366.949.935 | 1.346.522.548.919 |
| c_post_breast_cancer_tested_valcol | 1 | 2 | 3 | Increase | 1.013.930.689 | 1.366.131.373 | 352.200.684 | 1.240.453.217.894 |
| p_mut_presence_relatives | 0.144 | 0.18 | 0.216 | Decrease | 1.070.701.583 | 1.369.025.202 | 298.323.619 | 889.969.817.545 |
| p_well_OC_carriers_no_surgery | 0.01048 | 0.013 | 0.0148 | Decrease | 1.106.296.348 | 1.332.184.819 | 225.888.472 | 510.256.017.173 |
| p_well_BC_Mastec | 0 | 0 | 0.09 | Increase | 1.190.031.031 | 139.391.684 | 203.885.809 | 415.694.229.988 |
| start_age | 30 | 30 | 40 | Decrease | 1.006.313.318 | 1.190.031.031 | 183.717.713 | 337.521.981.482 |
| u_health_neg_test_valcol | 1 | 2 | 3 | Increase | 110.954.746 | 1.283.103.918 | 173.556.458 | 301.218.441.121 |
| u_prof_both_valcol | 1 | 2 | 3 | Decrease | 1.123.843.242 | 1.264.502.848 | 140.659.606 | 197.851.247.465 |
| p_bilateral_mastectomy | 0.02065 | 0.03 | 0.04528 | Decrease | 1.117.479.984 | 1.238.856.205 | 121.376.221 | 147.321.870.605 |
| u_health_high_risk_valcol | 1 | 2 | 3 | Decrease | 1.132.834.629 | 1.253.310.199 | 12.047.557 | 145.143.629.501 |
| HR_SOP_overall_mortality | 0 | 0.4 | 0.4 | Increase | 108.866.697 | 1.190.031.031 | 101.364.061 | 102.746.728.402 |
| u_prof_sop_health_valcol | 1 | 2 | 3 | Decrease | 1.145.648.094 | 123.799.139 | 92.343.297 | 85.272.844.747 |
| p_salpingo_ooforectomy | 0.1 | 0.12 | 0.14 | Decrease | 1.148.731.638 | 1.234.823.813 | 86.092.175 | 74.118.625.624 |
| u_prof_mast_health_valcol | 1 | 2 | 3 | Decrease | 115.481.416 | 1.227.463.391 | 72.649.231 | 52.779.107.654 |
| p_OC_death_SOP | 0 | 0.09508 | 0.09508 | Increase | 1.010.656.626 | 1.077.076.316 | 6.641.969 | 44.115.752.052 |
| c_profilatic_bilateral_mastectomy | 0 | 3484.26 | 3484.26 | Increase | 1.127.709.071 | 1.190.031.031 | 6.232.196 | 38.840.266.809 |
| c_both | 2463.16 | 4105.26 | 5747.36 | Increase | 1.166.533.613 | 1.213.528.449 | 46.994.835 | 22.085.145.346 |
| u_annual_increase_after_both_valcol | 1 | 2 | 3 | Decrease | 1.169.382.238 | 1.211.422.159 | 42.039.921 | 17.673.549.311 |
| c_post_ovarian_cancer_valcol | 1 | 2 | 3 | Increase | 1.171.602.138 | 1.209.238.731 | 37.636.593 | 14.165.130.958 |
| p_well_death_SOP | 0 | 0.004 | 0.004 | Increase | 108.866.697 | 1.125.084.846 | 36.417.876 | 13.262.617.026 |
| p_Well_BC_carriers_no_surgery_valcol | 1 | 2 | 3 | Decrease | 1.172.673.368 | 1.207.262.032 | 34.588.664 | 11.963.757.059 |
| p_BC_or_Post_BC_OC | 0.004 | 0.007 | 0.01 | Decrease | 1.173.610.762 | 1.208.095.303 | 34.484.541 | 11.891.835.808 |
| p_BC_or_Post_BC_Metastatic_BC | 0.0097 | 0.0134 | 0.01737 | Decrease | 1.174.031.917 | 1.206.683.829 | 32.651.912 | 10.661.473.395 |
| HR_SOP_OC_mortality | 0.168 | 0.21 | 0.252 | Increase | 1.173.149.399 | 1.203.154.007 | 30.004.608 | 900.276.503 |
| c_health_no_carriers_valcol | 1 | 2 | 3 | Decrease | 117.858.598 | 1.201.476.082 | 22.890.103 | 523.956.801 |
| p_well_death_both | 0 | 0.004 | 0.004 | Increase | 1.025.875.369 | 104.810.732 | 2.223.195 | 4.942.596.073 |
| HR_mastec_overall_mortality | 0.32 | 0.4 | 0.48 | Increase | 1.179.482.662 | 1.199.849.023 | 20.366.361 | 4.147.886.519 |
| p_well_death_mastec | 0 | 0.004 | 0.004 | Increase | 1.109.569.726 | 1.127.312.077 | 17.742.351 | 3.147.910.042 |
| c_profilatic_bilateral_salpingo_oophorectomy | 372.6 | 621 | 869.4 | Increase | 1.182.922.136 | 1.197.139.926 | 14.217.791 | 2.021.455.726 |
| u_post_ovarian_cancer_valcol | 1 | 2 | 3 | Decrease | 1.183.673.046 | 1.196.457.687 | 12.784.641 | 163.447.034 |
| HR_SOP_BC_mortality | 0 | 0.44 | 0.44 | Increase | 1.177.556.356 | 1.190.031.031 | 12.474.675 | 1.556.175.211 |
| p_OC_death_valcol | 1 | 2 | 3 | Increase | 1.186.421.735 | 1.196.657.329 | 10.235.594 | 1.047.673.919 |
| c_metast_breast_cancer | 9894.74 | 1.839.260.935 | 23087.72 | Decrease | 1.185.746.987 | 1.193.621.941 | 7.874.953 | 620.148.914 |
| c_metast_breast_cancer_valcol | 1 | 2 | 3 | Decrease | 118.623.218 | 1.193.829.882 | 7.597.702 | 577.250.774 |
| p_Well_BC_no_carriers_valcol | 1 | 2 | 3 | Decrease | 1.187.307.942 | 1.192.792.333 | 5.484.391 | 300.785.483 |
| p_BC_or_post_BC_death_SOP | 0.0048 | 0.006 | 0.0072 | Increase | 1.187.568.503 | 1.192.477.557 | 4.909.054 | 240.988.135 |
| p_BC_or_Post_BC_BC_carriers | 0.054 | 0.069 | 0.091 | Decrease | 1.187.282.062 | 1.191.837.479 | 4.555.417 | 207.518.205 |
| u_annual_increase_after_mastectomy_valcol | 1 | 2 | 3 | Decrease | 1.188.823.916 | 11.912.406 | 2.416.684 | 58.403.608 |
| u_annual_increase_after_BC_valcol | 1 | 2 | 3 | Increase | 1.189.662.267 | 1.190.400.023 | 737.756 | 5.442.844 |
| p_metastBC_Death_valcol | 1 | 2 | 3 | Increase | 1.189.684.331 | 1.190.345.475 | 661.144 | 4.371.112 |
| u_metast_breast_cancer_valcol | 1 | 2 | 3 | Increase | 1.189.913.537 | 1.190.148.548 | 235.011 | 5.523 |
| p_BC_or_Post_BC_BC_no_carriers | 0.00222 | 0.00366 | 0.00585 | Decrease | 1.189.956.833 | 1.190.079.524 | 12.269 | 150.529 |
| p_well_OC_no_carriers | 0.00007 | 0.00009 | 0.0001 | Increase | 1.190.020.902 | 1.190.041.156 | 0.20254 | 0.04102 |
| u_annual_increase_after_OC_valcol | 1 | 2 | 3 | Decrease | 1.190.029.528 | 1.190.032.533 | 0.03005 | 0.0009 |
| u_breast_cancer | 0.429 | 0.679 | 0.929 | Increase | 1.190.031.031 | 1.190.031.031 | 0 | 0 |
| u_ovarian_cancer | 0.47 | 0.52 | 0.57 | Increase | 1.190.031.031 | 1.190.031.031 | 0 | 0 |
| u_post_breast_cancer_valcol | 1 | 2 | 3 | Increase | 1.190.031.031 | 1.190.031.031 | 0 | 0 |
| p_well_death_valcol | 1 | 2 | 3 | Increase | 1.190.031.031 | 1.190.031.031 | 0 | 0 |
| u_breast_cancer_valcol | 0 | 2 | 3 | Increase | 1.190.031.031 | 1.190.031.031 | 0 | 0 |
| u_ovarian_cancer_valcol | 0 | 2 | 3 | Increase | 1.190.031.031 | 1.190.031.031 | 0 | 0 |
| p_BC_or_post_BC_death_mastec | 0 | 0.006 | 0.006 | Increase | 1.190.031.031 | 1.190.031.031 | 0 | 0 |
| HR_mastect_BC_mortality | 0 | 0.06 | 0.06 | Increase | 1.190.031.031 | 1.190.031.031 | 0 | 0 |
